# Supplementary material for: A Critical View of the Application of the APEX Software (Aqueous Photochemistry of Environmentally-Occurring Xenobiotics) to Predict Photoreaction Kinetics in Surface Freshwaters
Source: Molecules. 2019 Dec 18;25(1):9. doi: 10.3390/molecules25010009 (PMC7017383; doi:10.3390/molecules25010009)
Supplement: Supplementary file 1 [file molecules-25-00009-s001.zip › 3.2.4_gcc-4.4.0.zip!/3.2.4_gcc-4.4.0/3.2.4_gcc-4.4.0/doc/octave/html/interpreter/Timing-Utilities.html]

Timing Utilities - Untitled


Next: Filesystem Utilities,
Up: System Utilities


---

### 34.1 Timing Utilities

Octave's core set of functions for manipulating time values are
patterned after the corresponding functions from the standard C library.
Several of these functions use a data structure for time that includes
the following elements:

`usec`: Microseconds after the second (0-999999). `sec`: Seconds after the minute (0-61). This number can be 61 to account for leap seconds. `min`: Minutes after the hour (0-59). `hour`: Hours since midnight (0-23). `mday`: Day of the month (1-31). `mon`: Months since January (0-11). `year`: Years since 1900. `wday`: Days since Sunday (0-6). `yday`: Days since January 1 (0-365). `isdst`: Daylight Savings Time flag. `zone`: Time zone.

In the descriptions of the following functions, this structure is
referred to as a tm\_struct.

— Loadable Function: **time** ()  
> Return the current time as the number of seconds since the epoch. The
> epoch is referenced to 00:00:00 CUT (Coordinated Universal Time) 1 Jan
> 1970. For example, on Monday February 17, 1997 at 07:15:06 CUT, the
> value returned by `time` was 856163706.
>
>
>
> **See also:** strftime, strptime, localtime, gmtime, mktime, now, date, clock, datenum, datestr, datevec, calendar, weekday.

— Function File: t = **now** ()  
> Returns the current local time as the number of days since Jan 1, 0000.
> By this reckoning, Jan 1, 1970 is day number 719529.
>
> The integral part, `floor (now)` corresponds to 00:00:00 today.
>
> The fractional part, `rem (now, 1)` corresponds to the current
> time on Jan 1, 0000.
>
> The returned value is also called a "serial date number"
> (see `datenum`).
>
>
>
> **See also:** clock, date, datenum.

— Function File: **ctime** (t)  
> Convert a value returned from `time` (or any other non-negative
> integer), to the local time and return a string of the same form as
> `asctime`. The function `ctime (time)` is equivalent to
> `asctime (localtime (time))`. For example,
>
> ```
>           ctime (time ())
>                => "Mon Feb 17 01:15:06 1997\n"
> ```

— Loadable Function: **gmtime** (t)  
> Given a value returned from time (or any non-negative integer),
> return a time structure corresponding to CUT. For example,
>
> ```
>           gmtime (time ())
>                => {
>                      usec = 0
>                      year = 97
>                      mon = 1
>                      mday = 17
>                      sec = 6
>                      zone = CST
>                      min = 15
>                      wday = 1
>                      hour = 7
>                      isdst = 0
>                      yday = 47
>                    }
> ```
>
>
>
>
> **See also:** strftime, strptime, localtime, mktime, time, now, date, clock, datenum, datestr, datevec, calendar, weekday.

— Loadable Function: **localtime** (t)  
> Given a value returned from time (or any non-negative integer),
> return a time structure corresponding to the local time zone.
>
> ```
>           localtime (time ())
>                => {
>                      usec = 0
>                      year = 97
>                      mon = 1
>                      mday = 17
>                      sec = 6
>                      zone = CST
>                      min = 15
>                      wday = 1
>                      hour = 1
>                      isdst = 0
>                      yday = 47
>                    }
> ```
>
>
>
>
> **See also:** strftime, strptime, gmtime, mktime, time, now, date, clock, datenum, datestr, datevec, calendar, weekday.

— Loadable Function: **mktime** (tm\_struct)  
> Convert a time structure corresponding to the local time to the number
> of seconds since the epoch. For example,
>
> ```
>           mktime (localtime (time ()))
>                => 856163706
> ```
>
>
>
>
> **See also:** strftime, strptime, localtime, gmtime, time, now, date, clock, datenum, datestr, datevec, calendar, weekday.

— Function File: **asctime** (tm\_struct)  
> Convert a time structure to a string using the following five-field
> format: Thu Mar 28 08:40:14 1996. For example,
>
> ```
>           asctime (localtime (time ()))
>                => "Mon Feb 17 01:15:06 1997\n"
> ```
>
> This is equivalent to `ctime (time ())`.

— Loadable Function: **strftime** (fmt, tm\_struct)  
> Format the time structure tm\_struct in a flexible way using the
> format string fmt that contains % substitutions
> similar to those in `printf`. Except where noted, substituted
> fields have a fixed size; numeric fields are padded if necessary.
> Padding is with zeros by default; for fields that display a single
> number, padding can be changed or inhibited by following the %
> with one of the modifiers described below. Unknown field specifiers are
> copied as normal characters. All other characters are copied to the
> output without change. For example,
>
> ```
>           strftime ("%r (%Z) %A %e %B %Y", localtime (time ()))
>                => "01:15:06 AM (CST) Monday 17 February 1997"
> ```
>
> Octave's `strftime` function supports a superset of the ANSI C
> field specifiers.
>
> Literal character fields:
>
> `%`: % character. `n`: Newline character. `t`: Tab character.
>
> Numeric modifiers (a nonstandard extension):
>
> `- (dash)`: Do not pad the field. `_ (underscore)`: Pad the field with spaces.
>
> Time fields:
>
> `%H`: Hour (00-23). `%I`: Hour (01-12). `%k`: Hour (0-23). `%l`: Hour (1-12). `%M`: Minute (00-59). `%p`: Locale's AM or PM. `%r`: Time, 12-hour (hh:mm:ss [AP]M). `%R`: Time, 24-hour (hh:mm). `%s`: Time in seconds since 00:00:00, Jan 1, 1970 (a nonstandard extension). `%S`: Second (00-61). `%T`: Time, 24-hour (hh:mm:ss). `%X`: Locale's time representation (%H:%M:%S). `%Z`: Time zone (EDT), or nothing if no time zone is determinable.
>
> Date fields:
>
> `%a`: Locale's abbreviated weekday name (Sun-Sat). `%A`: Locale's full weekday name, variable length (Sunday-Saturday). `%b`: Locale's abbreviated month name (Jan-Dec). `%B`: Locale's full month name, variable length (January-December). `%c`: Locale's date and time (Sat Nov 04 12:02:33 EST 1989). `%C`: Century (00-99). `%d`: Day of month (01-31). `%e`: Day of month ( 1-31). `%D`: Date (mm/dd/yy). `%h`: Same as %b. `%j`: Day of year (001-366). `%m`: Month (01-12). `%U`: Week number of year with Sunday as first day of week (00-53). `%w`: Day of week (0-6). `%W`: Week number of year with Monday as first day of week (00-53). `%x`: Locale's date representation (mm/dd/yy). `%y`: Last two digits of year (00-99). `%Y`: Year (1970-).
>
>
>
>
> **See also:** strptime, localtime, gmtime, mktime, time, now, date, clock, datenum, datestr, datevec, calendar, weekday.

— Loadable Function: [tm\_struct, nchars] = **strptime** (str, fmt)  
> Convert the string str to the time structure tm\_struct under
> the control of the format string fmt.
>
> If fmt fails to match, nchars is 0; otherwise it is set to the
> position of last matched character plus 1. Always check for this unless
> you're absolutely sure the date string will be parsed correctly.
>
>
>
> **See also:** strftime, localtime, gmtime, mktime, time, now, date, clock, datenum, datestr, datevec, calendar, weekday.

Most of the remaining functions described in this section are not
patterned after the standard C library. Some are available for
compatibility with matlab and others are provided because they are
useful.

— Function File: **clock** ()  
> Return a vector containing the current year, month (1-12), day (1-31),
> hour (0-23), minute (0-59) and second (0-61). For example,
>
> ```
>           clock ()
>                => [ 1993, 8, 20, 4, 56, 1 ]
> ```
>
> The function clock is more accurate on systems that have the
> `gettimeofday` function.

— Function File: **date** ()  
> Return the date as a character string in the form DD-MMM-YY. For
> example,
>
> ```
>           date ()
>                => "20-Aug-93"
> ```

— Function File: **etime** (t1, t2)  
> Return the difference (in seconds) between two time values returned from
> `clock`. For example:
>
> ```
>           t0 = clock ();
>            many computations later...
>           elapsed_time = etime (clock (), t0);
> ```
>
> will set the variable `elapsed_time` to the number of seconds since
> the variable `t0` was set.
>
>
>
> **See also:** tic, toc, clock, cputime.

— Built-in Function: [total, user, system] = **cputime** ();  
> Return the CPU time used by your Octave session. The first output is
> the total time spent executing your process and is equal to the sum of
> second and third outputs, which are the number of CPU seconds spent
> executing in user mode and the number of CPU seconds spent executing in
> system mode, respectively. If your system does not have a way to report
> CPU time usage, `cputime` returns 0 for each of its output values.
> Note that because Octave used some CPU time to start, it is reasonable
> to check to see if `cputime` works by checking to see if the total
> CPU time used is nonzero.

— Function File: **is\_leap\_year** (year)  
> Return 1 if the given year is a leap year and 0 otherwise. If no
> arguments are provided, `is_leap_year` will use the current year.
> For example,
>
> ```
>           is_leap_year (2000)
>                => 1
> ```

— Built-in Function: **tic** ()  
— Built-in Function: **toc** ()  
> Set or check a wall-clock timer. Calling `tic` without an
> output argument sets the timer. Subsequent calls to `toc`
> return the number of seconds since the timer was set. For example,
>
> ```
>           tic ();
>           # many computations later...
>           elapsed_time = toc ();
> ```
>
> will set the variable `elapsed_time` to the number of seconds since
> the most recent call to the function `tic`.
>
> If called with one output argument then this function returns a scalar
> of type `uint64` and the wall-clock timer is not started.
>
> ```
>           t = tic; sleep (5); (double (tic ()) - double (t)) * 1e-6
>                => 5
> ```
>
> Nested timing with `tic` and `toc` is not supported.
> Therefore `toc` will always return the elapsed time from the most
> recent call to `tic`.
>
> If you are more interested in the CPU time that your process used, you
> should use the `cputime` function instead. The `tic` and
> `toc` functions report the actual wall clock time that elapsed
> between the calls. This may include time spent processing other jobs or
> doing nothing at all. For example,
>
> ```
>           tic (); sleep (5); toc ()
>                => 5
>           t = cputime (); sleep (5); cputime () - t
>                => 0
> ```
>
> (This example also illustrates that the CPU timer may have a fairly
> coarse resolution.)

— Built-in Function: **pause** (seconds)  
> Suspend the execution of the program. If invoked without any arguments,
> Octave waits until you type a character. With a numeric argument, it
> pauses for the given number of seconds. For example, the following
> statement prints a message and then waits 5 seconds before clearing the
> screen.
>
> ```
>           fprintf (stderr, "wait please...\n");
>           pause (5);
>           clc;
> ```

— Built-in Function: **sleep** (seconds)  
> Suspend the execution of the program for the given number of seconds.

— Built-in Function: **usleep** (microseconds)  
> Suspend the execution of the program for the given number of
> microseconds. On systems where it is not possible to sleep for periods
> of time less than one second, `usleep` will pause the execution for
> `round (`microseconds `/ 1e6)` seconds.

— Function File: **datenum** (year, month, day)  
— Function File: **datenum** (year, month, day, hour)  
— Function File: **datenum** (year, month, day, hour, minute)  
— Function File: **datenum** (year, month, day, hour, minute, second)  
— Function File: **datenum** (`"date"`)  
— Function File: **datenum** (`"date"`, p)  
> Returns the specified local time as a day number, with Jan 1, 0000
> being day 1. By this reckoning, Jan 1, 1970 is day number 719529.
> The fractional portion, p, corresponds to the portion of the
> specified day.
>
> Notes:
>
> - Years can be negative and/or fractional.- Months below 1 are considered to be January.- Days of the month start at 1.- Days beyond the end of the month go into subsequent months.- Days before the beginning of the month go to the previous month.- Days can be fractional.
>
> **Warning:** this function does not attempt to handle Julian
> calendars so dates before Octave 15, 1582 are wrong by as much
> as eleven days. Also be aware that only Roman Catholic countries
> adopted the calendar in 1582. It took until 1924 for it to be
> adopted everywhere. See the Wikipedia entry on the Gregorian
> calendar for more details.
>
> **Warning:** leap seconds are ignored. A table of leap seconds
> is available on the Wikipedia entry for leap seconds.
>
>
>
> **See also:** date, clock, now, datestr, datevec, calendar, weekday.

— Function File: str = **datestr** (date, [f, [p]])  
> Format the given date/time according to the format `f` and return
> the result in str. date is a serial date number (see
> `datenum`) or a date vector (see `datevec`). The value of
> date may also be a string or cell array of strings.
>
> f can be an integer which corresponds to one of the codes in
> the table below, or a date format string.
>
> p is the year at the start of the century in which two-digit years
> are to be interpreted in. If not specified, it defaults to the current
> year minus 50.
>
> For example, the date 730736.65149 (2000-09-07 15:38:09.0934) would be
> formatted as follows:
>
> | Code | Format | Example |
> | --- | --- | --- |
> | 0 | dd-mmm-yyyy HH:MM:SS | 07-Sep-2000 15:38:09 |
> | 1 | dd-mmm-yyyy | 07-Sep-2000 |
> | 2 | mm/dd/yy | 09/07/00 |
> | 3 | mmm | Sep |
> | 4 | m | S |
> | 5 | mm | 09 |
> | 6 | mm/dd | 09/07 |
> | 7 | dd | 07 |
> | 8 | ddd | Thu |
> | 9 | d | T |
> | 10 | yyyy | 2000 |
> | 11 | yy | 00 |
> | 12 | mmmyy | Sep00 |
> | 13 | HH:MM:SS | 15:38:09 |
> | 14 | HH:MM:SS PM | 03:38:09 PM |
> | 15 | HH:MM | 15:38 |
> | 16 | HH:MM PM | 03:38 PM |
> | 17 | QQ-YY | Q3-00 |
> | 18 | QQ | Q3 |
> | 19 | dd/mm | 13/03 |
> | 20 | dd/mm/yy | 13/03/95 |
> | 21 | mmm.dd.yyyy HH:MM:SS | Mar.03.1962 13:53:06 |
> | 22 | mmm.dd.yyyy | Mar.03.1962 |
> | 23 | mm/dd/yyyy | 03/13/1962 |
> | 24 | dd/mm/yyyy | 12/03/1962 |
> | 25 | yy/mm/dd | 95/03/13 |
> | 26 | yyyy/mm/dd | 1995/03/13 |
> | 27 | QQ-YYYY | Q4-2132 |
> | 28 | mmmyyyy | Mar2047 |
> | 29 | yyyymmdd | 20470313 |
> | 30 | yyyymmddTHHMMSS | 20470313T132603 |
> | 31 | yyyy-mm-dd HH:MM:SS | 1047-03-13 13:26:03 |
>
> If f is a format string, the following symbols are recognized:
>
> | Symbol | Meaning | Example |
> | --- | --- | --- |
> | yyyy | Full year | 2005 |
> | yy | Two-digit year | 2005 |
> | mmmm | Full month name | December |
> | mmm | Abbreviated month name | Dec |
> | mm | Numeric month number (padded with zeros) | 01, 08, 12 |
> | m | First letter of month name (capitalized) | D |
> | dddd | Full weekday name | Sunday |
> | ddd | Abbreviated weekday name | Sun |
> | dd | Numeric day of month (padded with zeros) | 11 |
> | d | First letter of weekday name (capitalized) | S |
> | HH | Hour of day, padded with zeros if PM is set | 09:00 |
> |  | and not padded with zeros otherwise | 9:00 AM |
> | MM | Minute of hour (padded with zeros) | 10:05 |
> | SS | Second of minute (padded with zeros) | 10:05:03 |
> | PM | Use 12-hour time format | 11:30 PM |
>
> If f is not specified or is `-1`, then use 0, 1 or 16,
> depending on whether the date portion or the time portion of
> date is empty.
>
> If p is nor specified, it defaults to the current year minus 50.
>
> If a matrix or cell array of dates is given, a vector of date strings is
> returned.
>
>
>
> **See also:** datenum, datevec, date, clock, now, datetick.

— Function File: v = **datevec** (date)  
— Function File: v = **datevec** (date, f)  
— Function File: v = **datevec** (date, p)  
— Function File: v = **datevec** (date, f, p)  
— Function File: [y, m, d, h, mi, s] = **datevec** (...)  
> Convert a serial date number (see `datenum`) or date string (see
> `datestr`) into a date vector.
>
> A date vector is a row vector with six members, representing the year,
> month, day, hour, minute, and seconds respectively.
>
> f is the format string used to interpret date strings
> (see `datestr`).
>
> p is the year at the start of the century in which two-digit years
> are to be interpreted in. If not specified, it defaults to the current
> year minus 50.
>
>
>
> **See also:** datenum, datestr, date, clock, now.

— Function File: d = **addtodate** (d, q, f)  
> Add q amount of time (with units f) to the datenum, d.
>
> f must be one of "year", "month", "day", "hour", "minute", or
> "second".
>
>
>
> **See also:** datenum, datevec.

— Function File: **calendar** (...)  
— Function File: c = **calendar** ()  
— Function File: c = **calendar** (d)  
— Function File: c = **calendar** (y, m)  
> If called with no arguments, return the current monthly calendar in
> a 6x7 matrix.
>
> If d is specified, return the calendar for the month containing
> the day d, which must be a serial date number or a date string.
>
> If y and m are specified, return the calendar for year y
> and month m.
>
> If no output arguments are specified, print the calendar on the screen
> instead of returning a matrix.
>
>
>
> **See also:** datenum.

— Function File: [n, s] = **weekday** (d, [form])  
> Return the day of week as a number in n and a string in s,
> for example `[1, "Sun"]`, `[2, "Mon"]`, ..., or
> `[7, "Sat"]`.
>
> d is a serial date number or a date string.
>
> If the string form is given and is `"long"`, s will
> contain the full name of the weekday; otherwise (or if form is
> `"short"`), s will contain the abbreviated name of the weekday.
>
>
>
> **See also:** datenum, datevec, eomday.

— Function File: e = **eomday** (y, m)  
> Return the last day of the month m for the year y.
>
>
>
> **See also:** datenum, datevec, weekday.

— Function File: **datetick** (form)  
— Function File: **datetick** (axis, form)  
— Function File: **datetick** (..., "keeplimits")  
— Function File: **datetick** (..., "keepticks")  
— Function File: **datetick** (...ax, ...)  
> Adds date formatted tick labels to an axis. The axis the apply the
> ticks to is determined by axis that can take the values "x",
> "y" or "z". The default value is "x". The formatting of the labels is
> determined by the variable form, that can either be a string in
> the format needed by `dateform`, or a positive integer that can
> be accepted by `datestr`.
>
>
>
> **See also:** datenum, datestr.
